# Xiao-Yao-San Formula Improves Cognitive Ability by Protecting the Hippocampal Neurons in Ovariectomized Rats

**DOI:** 10.1155/2020/4156145

**Published:** 2020-06-19

**Authors:** Lina Liu, Fei Ge, Haoran Yang, Huilian Shi, Weiting Lu, Zhiguang Sun, Jing Yan, Fei Qiao

**Affiliations:** ^1^Department of Hepatology, The Affiliated Hospital of Nanjing University of Chinese Medicine, Nanjing 210029, China; ^2^First Clinical Medical College, Nanjing University of Chinese Medicine, Nanjing 210023, China; ^3^Department of Gastroenterology, Haian Hospital of Traditional Chinese Medicine, Hainan 226600, China; ^4^Key Laboratory for Metabolic Diseases in Chinese Medicine, First Clinical Medical College, Nanjing University of Chinese Medicine, Nanjing 210023, China

## Abstract

Xiao-Yao-San (XYS) decoction is a traditional Chinese medicine formula. This study aimed to investigate the effect of XYS on cognitive abilities and its underlying mechanism in ovariectomized rats. Female Sprague-Dawley rats were ovariectomized and treated with XYS (3 g/kg or 9 g/kg) by gavage, with subcutaneous injection of 17-*β* estradiol (E2, 2 *μ*g/kg) as a positive drug control and gavage of 1 ml saline (0.9%) as a placebo control. After 6 weeks of treatment, rats were examined using the Morris water maze test. The estradiol level in the serum and hippocampus was measured by ELISA. Golgi staining was performed to observe neuronal morphology in the hippocampus. Apoptosis of hippocampal cells was observed by TUNEL staining. The protein content of *N*-methyl-D-aspartate receptor (NMDAR) 2A and 2B in the hippocampal CA1 region was determined by Western blot and immunohistochemistry. Expression of estrogen receptor (ER) and PI3K signaling was detected by Western blot. Compared with the sham group, both learning and memory were impaired in ovariectomized rats. Rats treated with E2 or high-dose XYS showed better learning and memory compared with the saline-treated rats. High-dose XYS significantly reduced escape latency in the spatial acquisition trial; meanwhile, the cross times and duration in the probe quadrant were increased in the spatial probe trial. High-dose XYS promoted the de novo synthesis of E2 content in the hippocampus but had no significant effect on the serum E2 level. Golgi staining indicated that high-dose XYS could increase the branch number and density of dendritic spines in the hippocampal CA1 area. TUNEL staining showed that high-dose XYS alleviated ovariectomy-induced neuronal apoptosis. The expression level of NMDAR2A and NMDAR2B in hippocampal CA1 was upregulated by XYS treatment. The beneficial effect of XYS was through activating ER*α*-PI3K signaling. In conclusion, high-dose XYS treatment can improve the cognitive abilities of ovariectomized rats by protecting the hippocampal neurons and restoring the hippocampal E2 level.

## 1. Introduction

Perimenopausal women often suffer from anxiety and depression related with estrogen level fluctuation [1, 2]. In addition to anxiety and depression, forgetfulness is another common complaint in perimenopausal women. The Seattle Midlife Women's Health Study reported that 60% of middle-aged women noted memory deterioration [3]. The Study of Women's Health Across the Nation (SWAN) found that perimenopause was a dependent risk factor for self-reported forgetfulness in middle-aged women [4]. Because the brain is an important target of estrogen regulation, it is speculated that estrogen deficiency may be responsible for the cognitive impairment. However, results from observational studies on the influence on cognition are not fully consistent [5]. Moreover, effective therapeutic hormone interventions to improve cognitive performance are yet to be established [6]. Moreover, other factors, such as perceived stress, mood, and physical health, may also contribute to memory symptoms [7]. Ovarian estrogen production is more likely to impact memory in the case of abrupt decrease rather than natural transition [8, 9]. Contrary to the inconsistency in human studies, the findings in ovariectomized animals support the association of estrogen deficiency with cognitive changes and efficiency of hormone replacement therapy [10–12].

Supplementation with phytoestrogens is a popular alternative therapy to relieve the symptoms of menopause. Phytoestrogens, such as flavonoids, isoflavones, and lignans, have structure similar to steroidal estrogens and can play an estrogen-like role by activating estrogen receptors (ERs). A meta-analysis supported the efficiency of soy isoflavone supplementation in improving visual memory in healthy postmenopausal women [13]. The SWAN Phytoestrogen Ancillary Study showed that high isoflavone or lignin intakes could improve processing speed or verbal memory at a certain stage of menopause, although the benefit was also ethnicity-/race-related [14]. The effect of phytoestrogens on improving cognition is more obviously indicated in animal studies. A hydroalcoholic extract from *Fenugreek* seeds could attenuate ovariectomy- (OVX-) induced hippocampal neuron damage and improve learning and memory performance in a rat model [15, 16].

Xiao-Yao-San (XYS), which means happy and carefree powder in Chinese, is a traditional Chinese medicine formula (TCM) that is used to treat menopausal anxiety and depression. The formula consists of *Bupleuri radix* (Chaihu), *Angelicae radix* (Danggui), *Paeoniae radix alba* (Baishao), *Atractylodis rhizome macrocephalae* (Baizhu), *Poria cocos* (Fuling), *Zingiberis siccatum rhizoma* (Shengjiang), *Menthae haplocalycis* (Bohe), and *Glycyrrhizae radix* (Gancao). A systematic analysis including 26 randomized trials showed that XYS was superior to antidepressants, indicated by Hamilton depression scale scores, and could enhance the efficacy of antidepressants in improving self-rating depression scale scores [17]. Perimenopausal women with exophthalmia and palpitation could also benefit from XYS treatment [18, 19]. Some therapeutic actions of XYS on relieving menopausal syndrome can be ascribed to the presence of phytoestrogens. A number of studies have reported the presence of phytoestrogens in XYS. Ergosterol is found in *Poria cocos* (Fuling) [20], and *β*-sitosterol is known to be present in *Angelicae radix* (Danggui) [21]. Miller-Marini et al. developed an estrogen-chimeric receptor/Gal4-response element regulated/luciferase-reporter assay for detecting the presence of phytoestrogens in complex TCM formulas. They analyzed Bupleurum & Peony Formula, a modified formula of XYS with the same principal herbs and found measurable phytoestrogen content [22].

The presence of phytoestrogens in XYS suggests that XYS has the potential to treat cognitive impairment caused by estrogen deficiency. An animal study showed that XYS could attenuate chronic immobilization stress (CIS) induced learning and memory deficit [23]. At present, no study has evaluated the effect of XYS on improving cognitive impairment in perimenopausal women. Therefore, the current study investigated the effect of XYS on cognitive abilities and its underlying mechanism in OVX rats.

## 2. Materials and Methods

### 2.1. Animals and Treatment

In total, 60 female Sprague-Dawley rats were used in this study. The study protocol was approved by the Institutional Ethics Committee of Experimental Animal (approval number: ACU170802). Animals were housed with free access to water and standard diet under controlled temperature and humidity condition. The rats were randomly divided into 5 groups (12 rats/group): sham group, OVX + saline or O-saline group, OVX + 17-*β* estradiol (E2) or O-E2 group, OVX + XYS 9 g/kg or O-XYS9 group, and OVX + XYS 3 g/kg or O-XYS3 group. Rats underwent OVX operation under anesthesia as described preciously [24]. Briefly, a longitudinal incision was made in one-third portion of the trunk and 1-2 cm away from each side of the spine. The adipose tissue was gently pulled out with tweezers. The ovary was identified, and the uterine horns were ligated. The ovary was removed, and the abdominal incision was sutured. In the sham group, the abdomen was incised without OVX.

Treatment was initiated two weeks after the OVX operation and lasted for six weeks. The rats in the XYS treatment groups received XYS by gavage needle at a single daily dose of 9 g/kg or 3 g/kg. Subcutaneous injection of E2 (2 *μ*g/kg) was set as a positive drug control. Gavage of 1 ml saline (0.9%) was set as a placebo control. The XYS decoction was used in the form of an extract (1 g/ml, equivalent to 1 g crude herbs/ml), which was provided by the Affiliated Hospital of Nanjing University of Traditional Chinese Medicine. The XYS decoction formula was reported preciously [25]. The dose of XYS used in the clinic for perimenopausal women is 175 g crude herbs/60 kg, which is equivalent to 3.5 g/kg in rats. Referring to other experimental reports on the rat model, we set the low dose at 3 g/kg and the high dose at 9 g/kg [26–28]. E2 powder (Cat. no. E2758, Sigma-Aldrich, St. Louis, MO, USA) was dissolved in a small amount of ethanol, and then 20 *μ*g/ml stocking solution was prepared with 0.9% saline.

### 2.2. Morris Water Maze Test

The learning and memory function of rats was assessed using the Morris water maze (MWM) test [29]. The MWM experiment device is a round pool (200 cm in diameter) with 38–40 cm high water. The water temperature is kept constant at 20–22°C. The WMW test consists of three parts, namely, the spatial acquisition trial (lasting 5 days), the spatial probe trial, and the visible platform trial. In the spatial acquisition trial, rats were gently placed in a random quadrant of the water tank with the platform hidden 1.5 cm in the northeast quadrant below the water. Each rat was allowed to swim for up to 60 s to find the platform. Rats who failed to find the platform within 60 s were guided to it and the latency was recorded as 60 s. The escape latency was recorded by a video analysis system (ANY-maze Animal Behavior Analysis System, Stoelting Company, USA). Rats were trained 4 times per day. After 5 days of training, rats received the spatial probe trial with the platform removed from the northeast quadrant. Rats were placed in the same starting location in the northwest quadrant. The traveling traces of the rats searching for the platform in 120 s were recorded. The duration and the cross times in the target quadrant were calculated to assess the retention of spatial memory. The next day after the spatial probe trial, rats received the visible platform trial similar to the hidden platform training, in which the platform was raised 1.5 cm above the water surface and placed in the southwest quadrant. Each rat was released 4 times, and each time from a different entry point. The escape latency, swimming distance, and swimming speed were recorded.

### 2.3. Sample Collection

After finishing the MWM test, rats were thoracotomized and intubated into ascending aorta via left ventricle under anesthesia with sodium pentobarbital (40 mg/kg via intraperitoneal injection). Trunk blood was collected using serum separator tube for detection of E2. Hippocampi from six rats were separated from cerebral cortex and surrounding brain tissues, quickly removed, washed with cold PBS, and frozen in liquid nitrogen for detection of E2 and Western blot. Three unperfused brain tissues were immobilized in 4% paraformaldehyde for 24 h and used for Golgi staining and immunohistochemistry. The left three rats were transcardially perfused with cold saline, followed by 4% paraformaldehyde fixation for 1 h. Brains were then removed, fixed in 4% paraformaldehyde for 24 h, and embedded in paraffin for TUNEL staining.

### 2.4. E2 Detection

Blood samples were allowed to clot at room temperature and were centrifuged at 1000*g* for 15 min at 4°C to separate serum. Hippocampus tissues were weighed, homogenized in cold saline, and centrifuged at 3000*g* for 20 min to collect supernatant. E2 content in serum and hippocampus homogenate supernatant was detected by ELISA kit (Shanghai Enzyme-linked Biotechnology Co. Ltd., Shanghai, China).

### 2.5. Golgi Staining

The hippocampus was cut into small blocks and processed for Golgi staining as described [17]. Briefly, the hippocampus blocks were immersed in Golgi staining solution (Sinopharm Chemical Reagent Co., Ltd., Shanghai, China) for 14 days, during which the dye solution was replaced every 2-3 days. Tissues were dehydrated with 30% sucrose and sectioned at 100 *μ*m. Sections were processed with ethanol dehydration, clarified with xylene, and mounted onto gelatin-coated slides. Golgi staining was successfully performed in all samples. The morphology of neurons in the hippocampus was observed under light microscope (Nikon, Tokyo, Japan). The neurons in the CA1 region of the hippocampus were analyzed by using the Neuron J plugged in the Image J software. Five sections were selected for each rat, and dendritic spines with complete morphology were selected for each section [30]. The density and branch points of the dendritic spines were measured.

### 2.6. Immunohistochemistry

Expression of *N*-methyl-D-aspartate receptor (NMDAR) subunits 2A and 2B in the hippocampus was determined by immunohistochemistry according to the standard procedure [31]. The primary antibodies used were rabbit anti-NMDAR2A (1 : 200, Boster Biological Technology, Wuhan, China) and rabbit anti-NMDAR2B (1 : 400, Servicebio Technology Co., Ltd., Wuhan, China). The secondary antibody was HRP-conjugated anti rabbit IgG (Servicebio).

### 2.7. TUNEL Staining

The morphological changes in hippocampus were detected by TUNEL staining with a commercial kit (Roche, Mannheim, Germany) as instructed. The sections underwent DAB colorimetric reaction followed by hematoxylin staining. The nucleus of TUNEL-positive apoptotic cells was brown under light microscope. Five sections were selected for each rat and three nonrepetitive fields (400x magnification) were selected for each section. The percentage of TUNEL-positive cells in all cells was calculated.

### 2.8. Western Blot

The hippocampal CA1 brain part was rapidly extracted and cut into small sections according to the brain Atlas. Hippocampus tissues were incubated overnight with RIPA lysis buffer (containing 100 Mm PMSF, purchased from Servicebio). The cell debris was removed by centrifugation at 13000*g* for 10 min to collect the supernatant containing protein. The protein concentration was determined by using BCA protein assay kit (Pierce, Rockford, IL, USA). Protein lysates (30 *μ*g/lane) were separated by 10% SDS-PAGE and electroblotted onto PVDF membrane. After being blocked with 5% nonfat milk, the membrane was probed overnight with the following primary antibodies: rabbit anti-NMDAR2A (1 : 500, Millipore, Billerica, MA, USA), rabbit anti-NMDAR2B (1 : 2000, Millipore), rabbit anti-CYP19 (1 : 1000, Abcam, Cambridge, UK), rabbit anti-ER*α* (1 : 2000, Millipore), rabbit anti-phospho-ER*α* (Ser118, 1 : 2000, Millipore), rabbit anti-phosphatidylinositol 3-kinase (PI3K) 110*β* (1 : 1000, Millipore), rabbit anti-Bax (1 : 1000, Abcam), rabbit anti-Bcl-2 (1 : 1000, Abcam), and rabbit anti-GAPDH (1 : 1000, Bioworld Technology, Louis Park, MN, USA). GAPDH was detected as internal control. Then the membrane was probed with secondary horseradish peroxidase- (HRP-) conjugated goat anti-rabbit IgG (1 : 5000, Bioworld) for 1.5 h. The bands were visualized by chemiluminescence method. Mean optical density of protein bands was quantified by Image J software.

### 2.9. Statistical Analysis

The experimental data were analyzed by GraphPad Prism 5 software (San Diego, CA, USA). Numerical data were expressed as mean ± SD. Difference between two groups was compared by *t*-test. The results of hidden platform trial were analyzed by group × day repeated-measures ANOVA followed by Tukey's post hoc. Other multiple comparisons were analyzed by one-way ANOVA followed by Tukey's post hoc. *P* value < 0.05 was considered statistically significant.

## 3. Results

### 3.1. Xiao-Yao-San Decoction Improves Spatial Learning and Memory Abilities of Ovariectomized Rats

**Figure 1:**
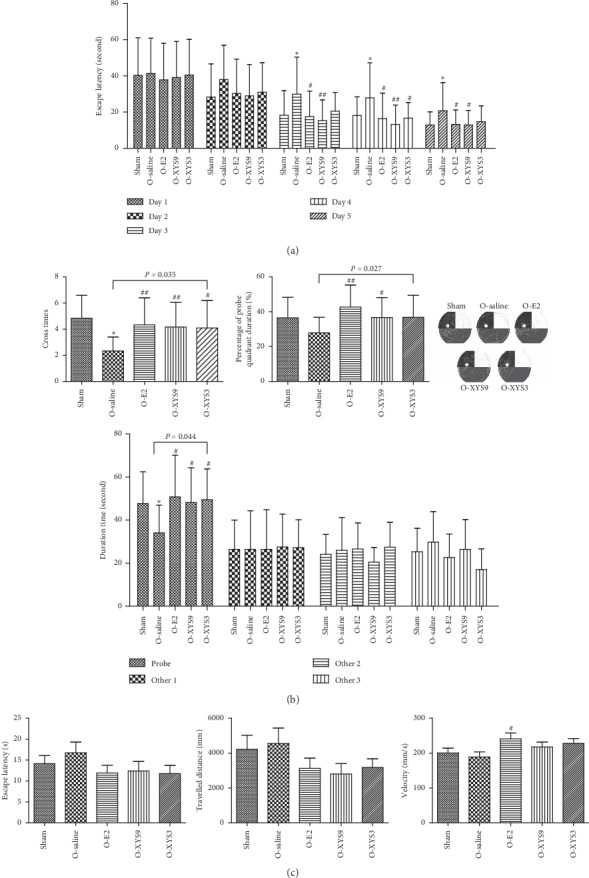
Effect of Xiao-Yao-San (XYS) on cognitive ability. (a) Spatial acquisition trial; (b) spatial probe trial; and (c) visible platform trial. O represents ovariectomy. ^*∗*^*P* < 0.05 versus sham; ^#^*P* < 0.05 versus O-saline; ^##^*P* < 0.01 versus O-saline. The results of each group represent average data from 12 animals.

Learning ability was first assessed using the spatial acquisition trial. Repeated-measures ANOVA showed that both treatment and training days affected the escape latency (Figure 1(a)). The escape latency in all groups decreased with the increase in training days (*P* < 0.001), and the difference was also significant in the trend of latency decline within each group (*P* < 0.001). There was no interaction between treatment and training days (*P* < 0.976). During the first two days of training, no significant difference was observed in the escape latency of each group. From the third day of training, the escape latency was significantly longer than that of the sham group; the escape latency of the E2 and high-dose XYS9 group was shorter than that of the saline group, but not different from that of the sham group. After 5 days of training, the rats were assessed with the probe trial to test the spatial memory. As shown in Figure 1(b), the cross times, duration time, and percentage of duration time in probe quadrant was significantly reduced in the saline group. No difference was observed in the duration time in other quadrants. Treatment with XYS or E2 could significantly restore the cross times and duration time in the probe quadrant to the level of the sham group. In order to exclude the effects of environmental factors and cognitive and activity levels of experimental animals on spatial learning and memory, rats also underwent the visible platform trial (Figure 1(c)). There was no significant difference in escape latency, swimming distance, and swimming velocity among the groups. Taken together, these results suggest that the spatial learning and memory were impaired in OVX rats, and high-dose XYS treatment could effectively improve the impaired cognitive functions.

### 3.2. Xiao-Yao-San Decoction Increases Hippocampal Estrogen Content in Ovariectomized Rats

**Figure 2:**
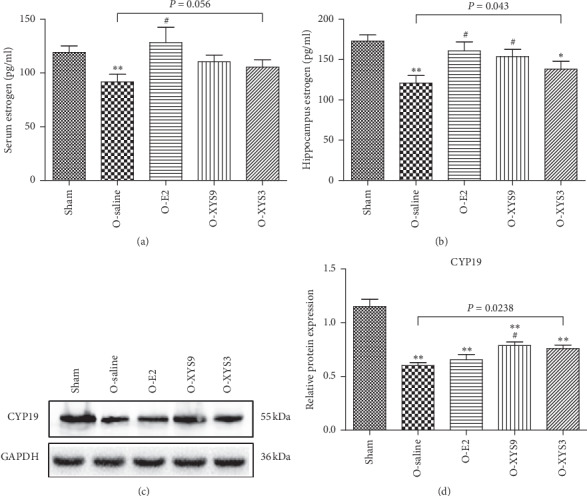
Effect of Xiao-Yao-San (XYS) on estradiol synthesis. The estradiol content in the serum (a) and hippocampus (b). (c-d) Relative expression of cytochrome P450 aromatase (CYP19) in the hippocampus. ^*∗*^*P* < 0.05 versus sham; ^*∗∗*^*P* < 0.01 versus sham; ^#^*P* < 0.05 versus O-saline. The results of each group represent average data from 12 animals.

Next, we studied the effect of XYS on estrogen level in OVX rats. As shown in Figure 2(a), XYS treatment did not improve the serum estradiol level reduced due to OVX; however, there was no significant difference in serum estradiol levels among the sham and OVX treatment groups. Of note, high-dose XYS and E2 increased hippocampal estradiol level as compared to saline solution (Figure 2(b)). O-XYS3 group still had lower hippocampal estradiol levels than the sham group. The change of hippocampal E2 content may not be explained by the change gonad-derived E2 content, but by the change of in situ synthesis. Therefore, we detected the expression of CYP19, the final enzyme in estrogen synthesis, in the hippocampus. The results showed that treatment with XYS or E2 could increase the expression of CYP19 compared with the saline group (*P*=0.0238), but only the difference between the high-dose XYS and saline groups was significant (*P* < 0.05). Treatment did not restore the CYP19 level to that of the sham group. The results suggest that XYS promoted the expression of aromatase in the hippocampus, thus increasing the de novo synthesis of E2.

### 3.3. Xiao-Yao-San Decoction Reduces Neuronal Damage in the Hippocampus of Ovariectomized Rats

**Figure 3:**
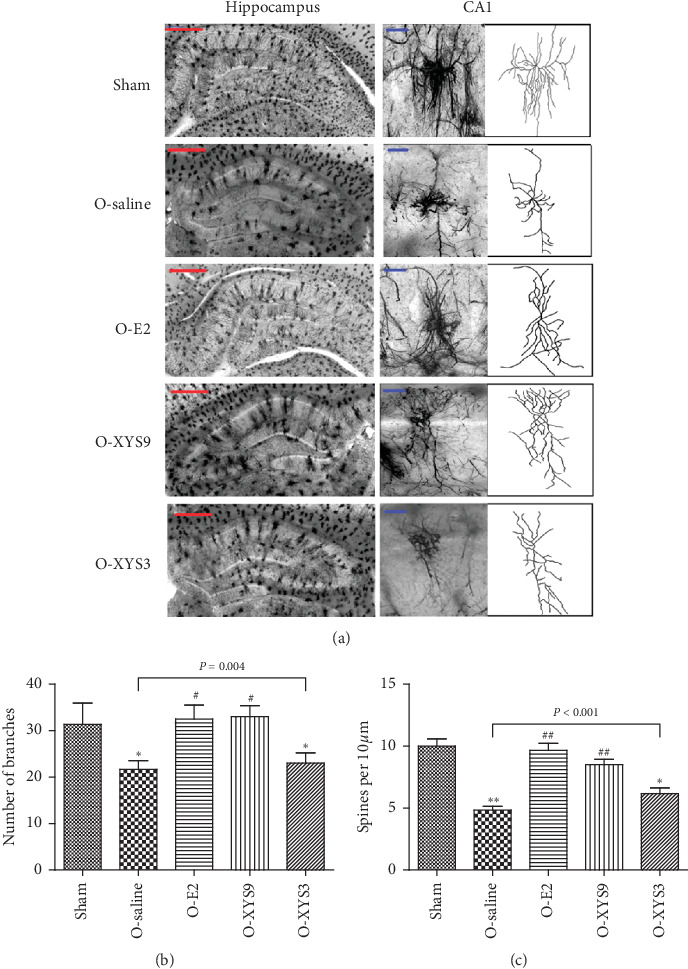
Effect of Xiao-Yao-San (XYS) on hippocampal CA1 dendritic arborization ((a), 50x magnification), CA1 branch points (b), and CA1 spine density (c). Red scale = 400 *μ*m; blue scale = 50 *μ*m. ^*∗*^*P* < 0.05 versus sham; ^*∗∗*^*P* < 0.01 versus sham; ^#^*P* < 0.05 versus O-saline; ^##^*P* < 0.01 versus O-saline. The results of each group represent average data from 3 animals.

**Figure 4:**
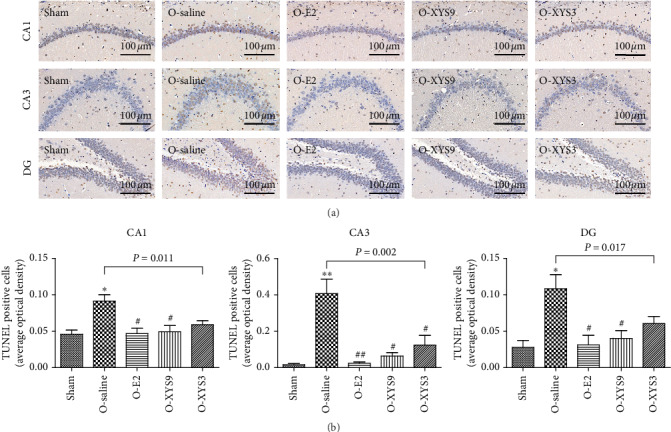
Effect of Xiao-Yan-San (XYS) on hippocampal apoptosis. (a) Representative TUNEL staining sections; (b) average optical density of TUNEL-positive cells in the CA1, CA3, and DG regions. ^*∗*^*P* < 0.05 versus sham; ^*∗∗*^*P* < 0.01 versus sham; ^#^*P* < 0.05 versus O-saline; ^##^*P* < 0.01 versus O-saline. The results of each group represent average data from 3 animals.

Cell morphology in the hippocampus, especially in the CA1 region, plays an important role in cognitive ability [32, 33]. The cell morphology in the hippocampus was analyzed by Golgi staining, as shown in Figure 3(a). Compared with the sham group, the number of neurons in the saline group decreased significantly, and the morphology of dendritic spines was damaged. Further analysis of CA1 neurons showed that the point number of neurons and dendritic spine density significantly decreased in the saline group (Figures 3(b) and 3(c)). High-dose XYS treatment increased the neuron number in the hippocampus and raised the point number of neurons and spine density in the CA1 region. E2 treatment had the same therapeutic effect as high-dose XYS. However, low-dose XYS did not effectively improve neuronal damage in the hippocampal CA1 region. Consistently, the apoptosis of hippocampal (CA1, CA3, DG) neurons was reduced by high-dose XYS or E2 treatment (Figure 4). However, low-dose XYS could only reduce the apoptosis of CA3 neurons but had no effect on CA1 and DG neurons. Taken together, high-dose XYS could reduce the neuronal damage in the hippocampus, especially in the CA1 region.

Xiao-Yao-San decoction increases hippocampal expression of NMDAR2A and NMDAR2B in ovariectomized rats.

**Figure 5:**
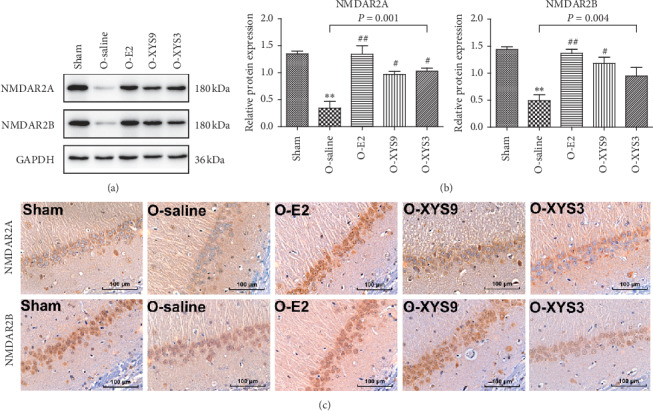
Effect of Xiao-Yan-San (XYS) on expression of NMDAR2A and NMDAR2B in the hippocampus. (a-b) Protein expression detected by Western blot. (c) Representative immunohistochemistry sections for NMDAR2A and NMDAR2B. ^*∗∗*^*P* < 0.01 versus sham; ^#^*P* < 0.05 versus O-saline; ^##^*P* < 0.01 versus O-saline. The results of each group represent average data from 3 animals.

Synaptic plasticity is the neurobiological basis for long-term learning and memory, and performance in the MWM test is influenced by NMDAR function [34]. Therefore, we further estimated the expression of NMDAR subunits 2A and 2B in the hippocampal CA1. Western blot (Figures 5(a) and 5(b)) showed that the expression of NMDAR2A and NMDAR2B was reduced in OVX rats. Treatment with XYS or E2 significantly increased the expression level of these two proteins to the same level as that in the sham group. Immunohistochemistry (Figure 5(c)) also showed that the proportion of positive stained cells increased in the treatment groups. Compared with the saline group, high-dose XYS significantly increased the expression of both NMDAR2A and NDMAR2B (*P*=0.009 and 0.013, resp.), but low-dose XYS could only significantly increase the expression of NMDAR2A (*P*=0.007).

### 3.4. Xiao-Yao-San Decoction Activates PI3K Signaling Pathway through ER*α*

**Figure 6:**
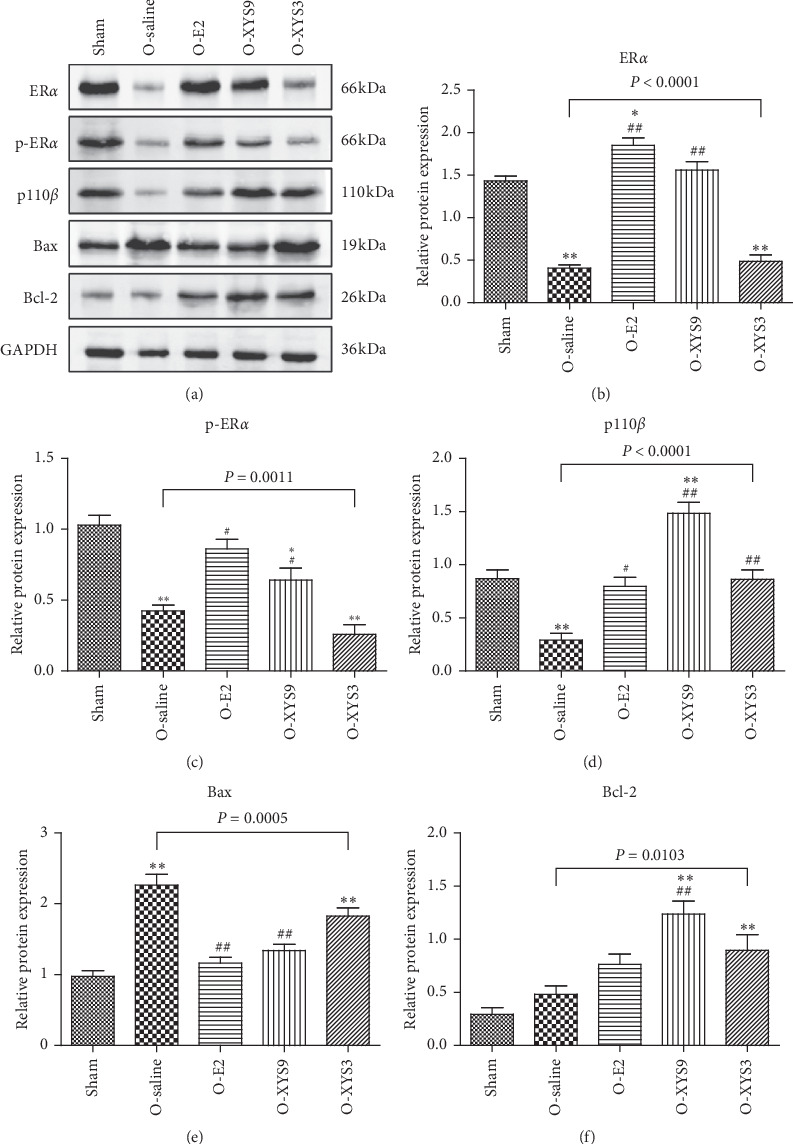
Effect of Xiao-Yao-San (XYS) on activation of p38 mitogen-activated protein kinase (MAPK) signaling pathway in the hippocampus. Protein expression of estrogen receptor *α* (ER*α*), phosphor-ER*α* (Ser118, p-ER*α*), phosphatidylinositol 3-kinase p110*β*, Bax, and Bcl-2 was detected by Western blot. ^*∗*^*P* < 0.05 versus sham; ^*∗∗*^*P* < 0.01 versus sham; ^#^*P* < 0.05 versus O-saline; ^##^*P* < 0.01 versus O-saline. The results of each group represent average data from 3 animals.

The impact of E2 or phytoestrogens on physiology is mediated by ER signaling. Studies have shown that the activation of extracellular signal-regulated kinase (ERK), a mitogen-activated protein kinase, is necessary for the beneficial effects of E2 on memory [35]. The phosphorylation of ERK1/2 is dependent on the activation of PI3K [36, 37]. Therefore, we investigated the effect of XYS on ER-PI3K signaling. As shown in Figure 6, high-dose XYS and E2 significantly promoted the expression of ER*α*, phosphorylated ER*α* (Ser118), and PI3K p110*β* (the catalytic subunit) compared with the saline group. Activation of ER*α*-PI3K further reduced the expression of proapoptotic factor Bax and enhanced the expression of the antiapoptotic factor Bcl-2. However, low-dose XYS did not significantly influence ER*α*-PI3K signaling, which is consistent with the results of Figures 3 and 4.

## 4. Discussion

XYS has long been used to treat perimenopausal anxiety and depression. Our study explored the new application of XYS in the treatment of OVX-induced cognitive impairment. The results showed that XYS effectively improved spatial learning and spatial memory in OVX rats. The possible mechanism is that XYS promotes the de novo synthesis of estrogen in the hippocampus, thus activating ER*α*-PI3K signaling pathway and inhibiting the apoptosis of hippocampal neurons.

The underlying mechanism of XYS for the treatment of depression has been extensively studied. XYS plays an antidepressant role by regulating a comprehensive network involving neurotransmitters, neurotrophins, hypothalamic-pituitary-adrenal axis, amino acids, and lipids [28, 38–44]. Some of these studies have shown that XYS can act on brain changes related with stress. In vitro assays showed that an XYS-containing serum could reverse the change in mitochondrial membrane potential, free calcium concentration, and apoptotic rate of neurons induced by oxidative stress [45]. Our study showed that XYS also attenuated neuronal apoptosis in the hippocampus region of OVX rats. The antiapoptotic effects of XYS suggest that it had the potential to act on a variety of pathological processes related to brain neuronal apoptosis.

The results of our study showed that XYS could restore the estrogen level in the hippocampus of OVX rats. The hippocampus is essential for memory formation and is rich in ERs, and estrogen facilitates higher cognitive and synaptic health via these ERs [46, 47]. Several clinical trials indicated the effectiveness of hormone replacement therapy on cognitive symptoms related to estrogen loss in women [48]. XYS could increase the estradiol level in the hippocampus, which should be the crucial basis for its effectiveness in improving the cognitive abilities of OVX rats. However, XYS did not increase the serum estradiol level. This may be because estradiol can be synthesized de novo in the hippocampus, while serum estradiol originates from the gonads [49]. Our results showed that XYS increased the expression of CYP19 in the hippocampus, suggesting that the increased E2 in the brain was likely to be hippocampus-derived [50]. The results of our study showed that XYS upregulated expression of NMDAR2A and NMDAR2B in the hippocampus of OVX rats. This finding was consistent with previous studies. Estrogen was found to increase both dendritic spine density and synapse number in the hippocampus through modulating NMDAR functions in OVX rats [51]. Estrogen could also restore hippocampal neuron morphology in aged rats by restoring the NMDAR2B level to that seen in young level in the CA1 region; what is more, estrogen may affect the mobility of NMDAR2A and NMDAR2B across the synapse [52]. Activation of NMDAR in turn could contribute to the synthesis of hippocampal E2 by inducing Ca^2+^ influx [50].

In addition to NMDARs, XYS may regulate the expression of other cognition-related factors in the brain. Loss of postsynaptic density protein 95 (PSD-95), synaptophysin, and brain-derived neurotrophic factor (BDNF) in the hippocampus has been directly correlated with learning and memory deficits [53–56]. The effect of XYS in CIS rats has been related to promoting the expression of PSD-95 and synaptophysin in the hippocampus [57]. XYS has also been shown to upregulate BDNF in the frontal cortex, hippocampal CA1 region, and amygdala of CIS rats [23, 58]. During the CIS treatment process of XYS, the c-Jun N-terminal kinase (JNK) signaling pathway was inhibited in the hippocampus [59].

Although we have not specifically explored which component is the main active one of XYS on learning and memory, our results suggest that the main source of phytoestrogens in XYS is a candidate worthy of further investigation. The research of antidepressant can also provide us with some clues. Jiali Liu et al. discovered that *Bupleuri radix* (Chaihu) is the principle antidepression drug in the XYS prescription based on the serum pharmacochemical analysis of the petroleum ether fraction [60]. *In vitro* study showed that *Bupleuri radix* extract promoted proliferation of neuroblastoma cells through activating PI3K/AKt pathway [61]. The activation of PI3K was also observed in our study. .

Current clinical reports show that XYS has a significant antidepressant therapeutic effect, but there is no clear toxicological study on the toxicity and side effects of XYS in detail. Generally speaking, XYS can be considered as a prescription without too many side effects. XYS has several modified recipes, whose basic ingredients are the same as XYS. A recent meta-analysis assessed the efficacy and safety of modified XYS in the treatment of perimenopausal syndrome. The pooled results suggest that modified XYS might be more effective and safer for the treatment of perimenopausal syndrome compared with hormone replacement therapy [62]. However, due to the poor methodology of included studies, the efficacy and safety of XYS need more rigorous trials for confirmation. Using ^1^H nuclear magnetic resonance (NMR) method, the research group of Xiaoxia Gao reported that the petroleum ether fraction containing lipophilic components was the most effective fraction for treating depression [63]. They also reported that *Bupleuri Radix,* the principal antidepressant component, could produce more toxic effects in the liver or kidney of healthy rats than in rats with chronic unpredictable mild stress. The toxic effects were associated with increased bile acid concentration, facilitation of lysine degradation, and metabolic disruption of sphingolipid, glycerophospholipid, and fatty acids [60, 64]. This result was corroborated in a recent metabolomic study of patients with depression. XYS played an antidepressant role by regulating the synthesis of leucine, valine, and isoleucine and the metabolism of binary acid, fatty acid, argine, and proline [65]. These studies suggest that it is necessary to closely monitor the patient's status with timely adjustment of the drug dose to prevent toxic side effects.

## 5. Conclusions

In this study, we demonstrated that high-dose XYS treatment had similar efficacy as E2 in improving the cognitive abilities of OVX rats. The therapeutic effect was meditated by protecting the hippocampal neurons and restoring the hippocampal E2 level. ER*α*-PI3K signaling pathway was involved in the process. Our study suggests that XYS could be an option for treatment of perimenopausal cognitive impairment. Presently, XYS is mainly used in the treatment of clinical anxiety and depression. More clinical data are needed to verify its effect on improving cognitive function.

## Data Availability

The data used to support the findings of this study are available from the corresponding authors upon request.
